# Prevalence of malaria and *Schistosoma mansoni* coinfection in sub Saharan Africa: A systematic review and meta-analysis

**DOI:** 10.1016/j.parepi.2025.e00422

**Published:** 2025-03-13

**Authors:** Wagaw Abebe, Birhanu Kassanew, Tadesse Misganaw, Agenagnew Ashagre, Getinet Kumie, Marye Nigatie, Yalewayker Gashaw, Ephrem Tamrat, Dagmawi Woldesenbet, Alembante Bazezew

**Affiliations:** a Department of Medical Laboratory Science, College of Health Sciences, Woldia University, Woldia, Ethiopia; b Department of Medical Laboratory Sciences, College of Medicine and Health Sciences, Wachemo University, Hossana, Ethiopia

**Keywords:** Prevalence, Malaria, *Schistosoma mansoni*, Coinfection, Sub Saharan Africa

## Abstract

**Background:**

Malaria and schistosomiasis are two parasite illnesses that share transmission sites in distinct tropical climates. Malaria-schistosomiasis coinfection is widespread in Africa. Also, malaria and *Schistosoma mansoni* coinfection cause exacerbation of health consequences and co-morbidities. However, there is limited pooled data on the prevalence of malaria and *Schistosoma mansoni* coinfection in sub-Saharan Africa.

**Objective:**

This systemic review and meta-analysis aimed to assess the prevalence of malaria and *Schistosoma mansoni* coinfection in sub-Saharan Africa.

**Method:**

Systematic search on PubMed, Scopus, Google Scholar, and Science Direct was used to identify relevant studies following reviews and meta-analysis guidelines. A total of eighteen relevant articles on the prevalence of malaria and *Schistosoma mansoni* coinfection were identified for final systematic review and meta-analysis. Extracted data was analyzed using STATA software version 17.0. The absence or presence of publication bias was assessed using Egger's test. Heterogeneity across studies was checked by I^2^ statistics; if the I^2^ value was ≥50 %, significant heterogeneity was considered and subgroup analysis was done.

**Results:**

A total of 18 studies were included for this systematic review and meta-analysis. From this meta-analysis, the pooled prevalence of malaria and *Schistosoma mansoni* coinfection was 17.39 % (95 % CI: 5.94–28.84). There was significant heterogeneity in prevalence of coinfection, with I^2^ values greater than or equal to 99.97 % at *P* = 0.00. The subgroup analysis based on year of publication showed that the pooled prevalence of malaria and *Schistosoma mansoni* coinfection in studies conducted 2014–2018 was 20.73 % (95 % CI: 0.66–40.80), while it was 14.68 % (95 % CI: 1.02–28.34) in studies conducted 2019–2024. On the other hand, subgroup analysis on diagnostic techniques showed significant differences in the pooled prevalence of malaria and *Schistosoma mansoni* coinfection.

**Conclusions:**

This systematic review and meta-analysis showed that malaria and *Schistosoma mansoni* coinfection are prevalent in sub-Saharan Africa.This highlights the region's major challenges in controlling malaria and *Schistosoma mansoni* coinfections.To ensure the efficiency of coinfections control and treatment, regular monitoring, identification, and reduction of the prevalence of malaria and *Schistosoma mansoni* coinfection must be maintained. Furthermore, cooperative efforts at local, countrywide, and global levels are necessary to address the multifaceted factors causal to malaria-*S.mansoni* coinfection.

## 1 Introduction

Malaria is a significant public health concern that continues to cause morbidity and mortality (Talapko et al., 2019). It is caused by *Plasmodium* protozoan parasites, which are spread by female *Anopheles* mosquitoes (Sinden and Gilles, 2017). Human malaria is caused by *Plasmodium falciparum*, *Plasmodium vivax*, *Plasmodium ovale*, and *Plasmodium malariae*, among which *P. falciparum* is primarily responsible for the majority of malaria-related fatalities and serious diseases *(*Tseha*, 2021**)*. It has recently been found that *P. knowlesi*, which causes malaria in macaque monkeys, may infect people in Southeast Asia (White, 2008). Malaria caused approximately half a million (429000) fatalities in 2016, with Sub-Saharan African nations accounting for more than 92 % of fatalities (Saleh et al., 2019). According to data from 85 malaria endemic countries, the global malaria burden increased from 227 million cases in 2019 to 241 million cases in 2020, with the majority of the increase coming from countries in the African region (Patel et al., 2024).

The malaria parasite causes the majority of the clinical indications of this disease during its asexual multiplication in red blood cells. Malaria's common clinical symptoms include fever, nausea, and headache, which may be followed by diarrhea and vomiting (Zambare et al., 2019). If the disease persists untreated, life-threatening consequences, generally known as severe malaria, may occur in *P. falciparum* infections (Bell and Molyneux, 2007). Severe malaria encompasses any of the following manifestations: cerebral malaria, severe anemia, renal failure, pulmonary edema, hypoglycemia, circulatory collapse, spontaneous bleeding, frequent generalized convulsions, acidosis, and hemoglobinuria (Singhal, 2004).

Schistosomiasis is one of the water-borne illnesses considered as water-based neglected tropical illnesses, which affect hundreds of millions of people, mainly in Sub-Saharan Africa (Bekana et al., 2021). It is an acute and chronic disease caused by trematode platyhelminths of the genus *Schistosoma (*Horak et al., *2014**)*. The *Schistosoma* species that infect humans include *Schistosoma mansoni*, *Schistosoma haematobium*, and *Schistosoma japonicum* (Gryseels et al., 2006). The clinical appearance and pathology of schistosomiasis are influenced by the *Schistosoma* species that causes disease, where it lives, and the degree of infection (Aagaard-Hansen, 2008). *Schistosoma mansoni* inhabits the mesenteric plexus, which can result in intestinal or hepatosplenic schistosomiasis, which affects the intestines, liver, and spleen (Colley et al., 2014). The main factor of *Schistosoma* pathogenesis is the host's immunological reaction to the antigens on the eggs, which leads to the development of granulomas in the liver and intestine where the eggs are lodged. This causes a cellular, granulomatous response to form, which leads to fibrosis and the most severe infection-related illness symptoms *(*Bindseil et al., *2004**)*.

According to a study about the global burden of disease, schistosomes infect 252,000,000 individuals worldwide, 90 % of whom live in sub-Saharan Africa, and are thought to have cost the global community 3,300,000 years of life with a handicap (Hotez et al., 2014). Similarly, according to World Health Organization (WHO, G.S, 2014), 258,000,000 people globally needed schistosomiasis preventative therapy on a frequent and regular basis (WHO, 2015). Several factors contribute to the continual and persistent spread of schistosomiasis in sub-Saharan Africa. These include climate change and global warming, closeness to water sources, irrigation and dam development, as well as socio-economic issues such as occupation and poverty (Adenowo et al., 2015).

Malaria and schistosomiasis are two parasite illnesses that share transmission sites in distinct tropical climates. Malaria-schistosomiasis co-infection is widespread in Africa. Furthermore, co-infections of these parasites are frequent as a result of geographical overlap between schistosomiasis and malaria, resulting in various types of association, aggravated health problems, and co-morbidities (Mwangi et al., 2006). Furthermore, coinfection has a considerable influence on the control of inflammatory factors related to the course of these diseases and their relative morbidity (Le Hesran et al., 2004). Malaria and *S. mansoni* infections pose public health and socioeconomic development challenges (Dhanaraj et al., 2015). Furthermore, morbidity and mortality from malaria and *S. mansoni* continue to be serious global concerns. Coinfection with these parasites is common in Sub-Saharan Africa (SSA), where over 90 % of these illnesses occur due to a huge geographical overlap (Organization, W.H., 2016; Kamau et al., 2021a; Degarege et al., 2012).

The challenges of developing a highly effective malaria vaccine have generated interest in understanding the interactions between malaria and coendemic helminth infections, such as those caused by *Schistosoma*, that may impair vaccine efficacy by modulating host-immune responses to *Plasmodium* infection and treatment (Doumbo et al., 2014; Okosun, 2016).

The patterns and incidence of malaria and schistosomiasis, including coinfections, are heavily influenced by social, economic, and environmental factors, with poor and rural areas being the most affected. Furthermore, persons who engage in specific activities and occupations are at higher risk due to greater environmental exposures (Adenowo et al., 2015; Kamau et al., 2021b).

Therefore, an in-depth awareness of malaria epidemiology during *Schistosoma* coinfection is required to make informed judgments about effective schistosomiasis and malaria management strategies in Sub-Saharan Africa. Additionally, information on the prevalence and clinical consequences of schistosomiasis coinfection with malaria is required in order to improve clinical care and prevention of malaria, particularly in schistosomiasis co-endemic regions (Eyong et al., 2023). To the greatest extent of our knowledge, little study has been done on the aggregate incidence and contributing factors of malaria and *S. mansoni* coinfection in sub-Saharan Africa. Therefore, this systematic review and meta-analysis attempted to determine the pooled prevalence of malaria and *S. mansoni* coinfection in sub Saharan Africa countries between 2014 and 2024.

## 2 Method

### 2.1 Design and protocol registration

This review was performed following the Preferred Reporting Item for Systematic Review and Meta-analysis Protocol (PRISMA-P 2020) guideline (Page et al., 2021). The PRISMA flow chart's four steps, which illustrate the study selection procedure from the first identified records to the included studies, were recorded in the results. The review protocol was established prior to conducting a literature search and registered under the CRD42024580835 registration number in the International Prospective Register of Systematic Reviews (PROSPERO) database. A protocol that addressed the review questions was developed by establishing inclusion/exclusion criteria and outcomes of interest.

### 2.2 Database and search strategy

This systematic review's initial searches began on 30/8/2024, and the protocol was registered on 29/08/2024. This systematic review and meta-analysis covered studies published in English and conducted in sub-Saharan Africa between 2014 and August 30, 2024. An extensive literature search was carried out to search for studies regarding the prevalence of malaria and *S. mansoni* coinfection in a sub-Saharan African population with diverse study topics. Systematic searches of the gray and electronic literature were conducted. Data were retrieved using PubMed, Science Direct, Scopus, and Google Scholar. Search terms were used separately and in combination, with Boolean operators such as “OR” or “AND” Keywords used in Google Scholar to find relevant studies included the following: [Prevalence AND malaria OR *Schistosoma mansoni* OR coinfection “AND *Plasmodium falciparum*, OR *Plasmodium malariae* OR, *Plasmodium vivax* OR *Plasmodium ovale* OR “AND “sub Saharan Africa “2014–2024]. The full search strategies for PubMed, ‘Prevalence’ AND ‘malaria’ OR ‘*Schistosoma mansoni’* OR ‘coinfection’ OR ‘*Plasmodium falciparum’* OR ‘*Plasmodium’* OR ‘*Plasmodium malariae’* OR ‘*Plasmodium vivax’* OR ‘*Plasmodium ovale”* AND “sub saharan Africa countries” AND “2014–2024″. Also, the full search strategies for Science direct, “Prevalence”AND “malaria” OR “*Schistosoma mansoni*” OR “coinfection” AND “sub saharan Africa countries” AND “2014–2024″. Similarly, the full search strategies for Scopus, ‘Prevalence’ AND ‘malaria’OR ‘*Schistosoma mansoni*’ OR ‘coinfection’ OR”*Plasmodium falciparum*” OR “*Plasmodium*” OR “*Plasmodium malariae*” OR, “*Plasmodium vivax*” OR “*Plasmodium ovale*” AND ‘sub saharan Africa countries” AND “2014–2024″. Furthermore, the full search strategies for Google Scholar, ‘Prevalence’ OR ‘malaria’ AND ‘*Schistosoma mansoni*’ AND ‘coinfection’ AND ‘sub saharan Africa countries” AND “2014–2024″. Furthermore, a snowball search was performed on the citation lists of the included studies. The keywords/strings were rearranged into phrases related to the outcome of interest. Papers from the reference section and citation lists of complete texts, such as original research papers and reviews, were retrieved to boost the likelihood of retrieving more data. Different combinations were designed for each electronic database to reduce the number of results obtained while increasing the number of relevant research. The researcher incorporated studies recorded from 2014 up to the 30th of August 2024.

### 2.3 Eligibility criteria

Articles retrieved from the aforementioned databases were imported into EndNote version 20 reference management software (Tomson Reuters, New York, NY). For this systematic review and meta-analysis, the included studies were: 1) observational studies including cross-sectional studies, cohort studies (retrospective and prospective), and case-control studies that report a prevalence of either prevalence of malaria and *S. mansoni* coinfection in sub Saharan Africa countries 2) Articles published in peer-reviewed journals or gray literature; and (3) articles published in English from start to August 30, 2024. We excluded studies if 1) they were not fully accessible; 2) they possessed a poor quality score as per the stated criteria; 3) case series, letters, comments, and editorials; and/or failed to measure the desired outcome (*i.e.* prevalence of malaria and *S. mansoni* coinfection).

### 2.4 Outcome of interest

The main outcome of interest was the prevalence of malaria and *S. mansoni* coinfection in sub Saharan Africa countries reported in the original paper both as a percentage and as the number of cases (n)/total number of participants (N).

### 2.5 Study selection and quality assessment

Quality appraisal of the studies was conducted using Joanna Brigg's Institute quality appraisal criteria (JBI) and Studies with 50 % and above on the quality scale was considered to have good quality (Munn et al., 2015). Three independent reviewers (W.A, E.T, and Y.G) screened the titles identified in the abovementioned databases. Following this, four reviewers (W.A, A.A, D·W, and G.K) independently screened eligible studies for abstract. Finally, full-text screening was conducted by four reviewers (W.A, T.M, A.A, and D·W).

### 2.6 Data extraction

A standardized data extraction form in Microsoft Excel 2010 was used for obtaining or record pertinent information from each included potential research. The extraction process covered a wide range of domains, including study characteristics such as first author, year of publication, study population, study design, number of participants, study area/region, prevalence of malaria and *S. mansoni* coinfection in each study. Five reviewers looked over the extracted data for correctness and consistency (W.A, A.A, E.T, G.K, and A.B). The fifth reviewer was also engaged where necessary (B·K).

### 2.7 Statistical analysis

The extracted data was entered into Microsoft Excel and analyzed using STATA version 17 (Stata Corp. Stata Statistical Software; College Station, TX: Stata Corp LP). A random-effects model was used to get an overall summary estimate of prevalence across studies. Point estimate with a confidence interval of 95 % was utilized. The presence or absence of publication bias was determined using Egger's test. Inverse of variance (I^2^) statistics were used to assess study heterogeneity. Significant heterogeneity was defined as an I^2^ value of ≥50 %. To identify causes of heterogeneity in studies with significant differences (I^2^ ≥ 50 %), we conducted subgroup analyses.

## 3 Results

### 3.1 Number of articles searched in the included information database

**Fig. 1:**
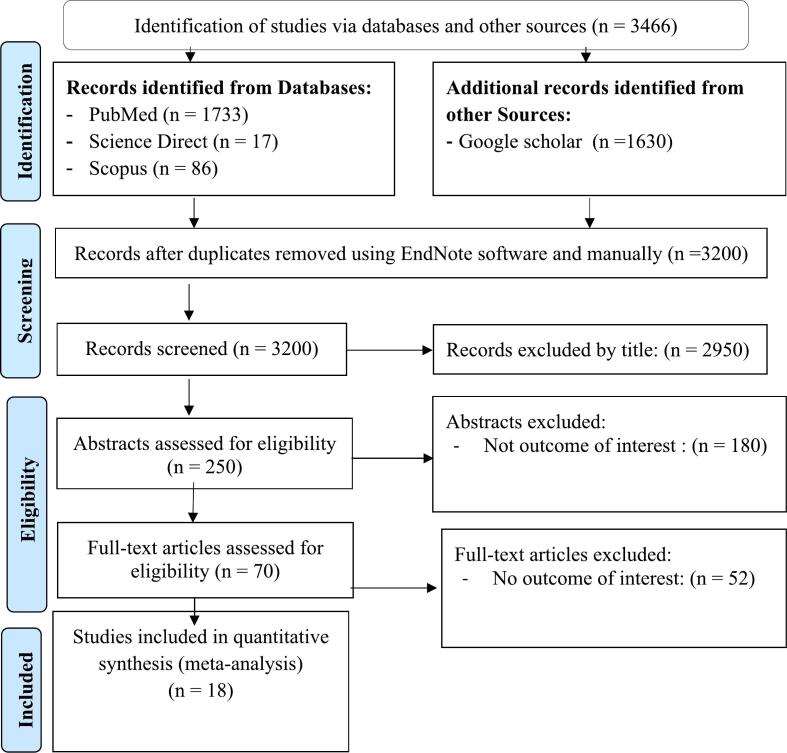
PRISMA flow diagram indicated the results of the search and reasons for exclusion (Page et al., 2021).

The electronic searches yielded a total of 3466 articles on prevalence of malaria and *S. mansoni* coinfection in sub Saharan Africa countries. A total of 3200 studies were identified, after which 266 duplicates were removed. A total of 3200 studies were screened to remove studies by title, abstract, and full-text articles, with 18 studies retained after the screening and eligibility process. Finally, 18 studies were included for both qualitative and quantitative analyses (Fig. 1).

### 3.2 Characteristics of the included studies

This study encompasses participants of all ages and genders. Also, from a total of 3466 studies, 18 articles were assessed (Kamau et al., 2021b; Dufera et al., 2016; Namulondo et al., 2024; Kinung'hi et al., 2014; Matangila et al., 2014; Afolabi et al., 2023; Nkemngo et al., 2022a; Hailu et al., 2018; Kinung'hi et al., 2017; Getie et al., 2015; Dassah et al., 2023; Lucia et al., 2015; Oboth et al., 2019; Echelibe et al., 2019; Jeannine et al., 2024; Wekesa, 2018; Akosah-Brempong et al., 2021; Mnkugwe et al., 2020). Each study was conducted using a cross-sectional study design, encompassing facility- and community-based studies.The prevalence of malaria-*S. mansoni* coinfection ranges from a minimum of 0.13 % (Wekesa, 2018) to a maximum of 88.4 % (Kinung'hi et al., 2017). Most studies were used microscopy technique for investigation of parasites (Dufera et al., 2016; Kinung'hi et al., 2014; Matangila et al., 2014; Hailu et al., 2018; Kinung'hi et al., 2017; Getie et al., 2015; Dassah et al., 2023; Lucia et al., 2015; Oboth et al., 2019; Jeannine et al., 2024; Wekesa, 2018; Akosah-Brempong et al., 2021) (Table 1).

**Table 1:** Summary of included studies in systematic review and meta-analysis.

Author	Year of Publication	Country	Study Design	Diagnostic Technique	Sample Size	Prevalence (%) of M and Sm Co	Quality
Mebrate D. *et al (*Dufera et al., *2016**)*	2016	Ethiopia	CS	Microscopy	810	12.1	9
Joyce N. *et al* (Namulondo et al., 2024)	2024	Uganda	CS	PCR	210	71	9
Safari M. et al. (Kinung'hi et al., 2014)	2014	Tanzania	CS	Microscopy	1546	27.2	9
Junior R. et al. (Matangila et al., 2014)	2014	Congo	CS	Microscopy	989	1.5	9
Muhammed O. et al. (Afolabi et al., 2023)	2023	Senegal	CS	PCR and Microscopy	910	0.7	9
Francis N. et al. (Nkemngo et al., 2022a)	2022	Cameroon	CS	RDT, Microscopy, and PCR	65	16.9	9
Tadesse H. et al. (Hailu et al., 2018)	2018	Ethiopia	CS	Microscopy	333	2.8	9
Safari M. et al. (Kinung'hi et al., 2017)	2017	Tanzania	CS	Microscopy	928	88.4	9
Sisay G. et al. (Getie et al., 2015)	2015	Ethiopia	CS	Microscopy	205	19.5	9
Sylvester D. et al. (Dassah et al., 2023)	2023	Ghana	CS	Microscopy	326	7.7	9
Edwin K. et al. (Kamau et al., 2021b)	2021	Kenya	CS	Microscopy and PCR	671	18	9
Nkengazong L. et al. (Lucia et al., 2015)	2015	Cameroon	CS	Microscopy	254	14.2	9
Paul O. et al. (Oboth et al., 2019)	2019	Uganda	CS	Microscopy	476	0.21	9
Hilda E. et al. (Echelibe et al., 2019)	2019	Cameroon	CS	RDT and Microscopy	149	30.2	9
UWIMANA J. et al. (Jeannine et al., 2024)	2024	Rwanda	CS	Microscopy	143	0.7	9
Antony W. et al. (Wekesa, 2018)	2018	Kenya	CS	Microscopy	750	0.13	9
G.Akosah-B. et al. (Akosah-Brempong et al., 2021)	2021	Ghana	CS	Microscopy	493	1.4	9
Rajabu H. et al. (Mnkugwe et al., 2020)	2020	Tanzania	CS	Microscopy and MRDT	824	1.6	9

Abbreviation: CS = Cross Sectional, M & Sm Co. = Malaria and *Schistosoma mansoni* Coinfection, MRDT = Malaria Rapid Diagnostic Test, PCR = Polymerase Chain Reaction, RDT = Rapid Diagnostic Test.

### 3.3 Heterogeneity and publication bias

The heterogeneity was evaluated for prevalence of malaria and *S. mansoni* Coinfection. There was significant heterogeneity in prevalence of malaria and *S. mansoni* coinfection, with I^2^ statistics indicating values greater than or equal to 99.97 % at *P* = 0.00. An Egger's test was used to evaluate the potential publication bias in the included studies. The result of Egger's test indicated that there was no publication bias with a *p*-value = 0.0825.

### 3.4 Prevalence of malaria and *S. mansoni* coinfection in sub Saharan Africa

**Fig. 2:**
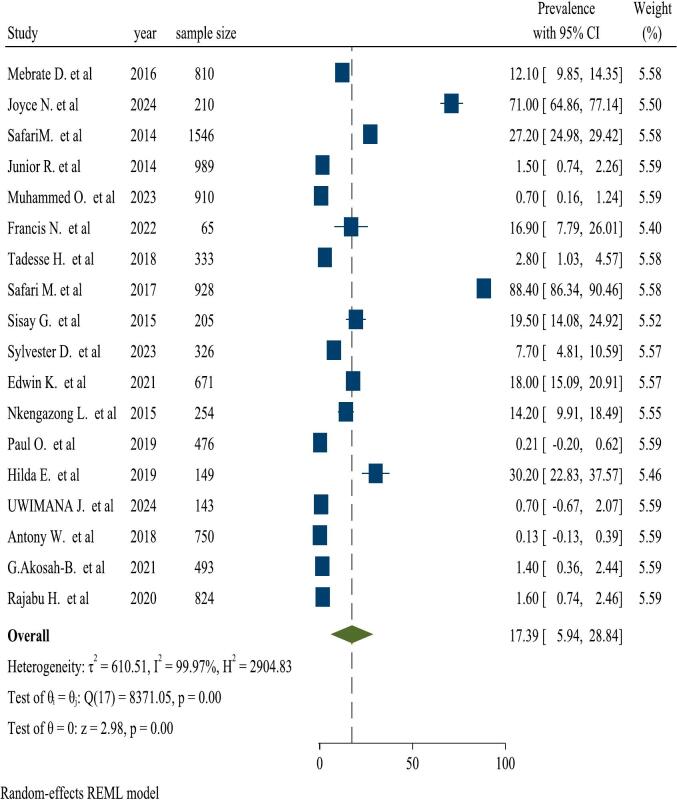
Forest plot showing the prevalence of malaria-*S. mansoni* coinfection.

The pooled prevalence of malaria and *S. mansoni* coinfection was 17.39 % (95 % CI: 5.94–28.84). A random-effects model shows the presence of heterogeneity among the included studies with 95 % CI (I^2^ = 99.97 % and *P*-value = 0.00). Due to the presence of significant heterogeneity between the included studies, subgroup analysis was carried out to know the prevalence of malaria and *S. mansoni* coinfection among articles (Fig. 2).

### 3.5 Subgroup analysis of malaria and *S. mansoni* coinfection by year of publication and diagnostic technique

**Fig. 3:**
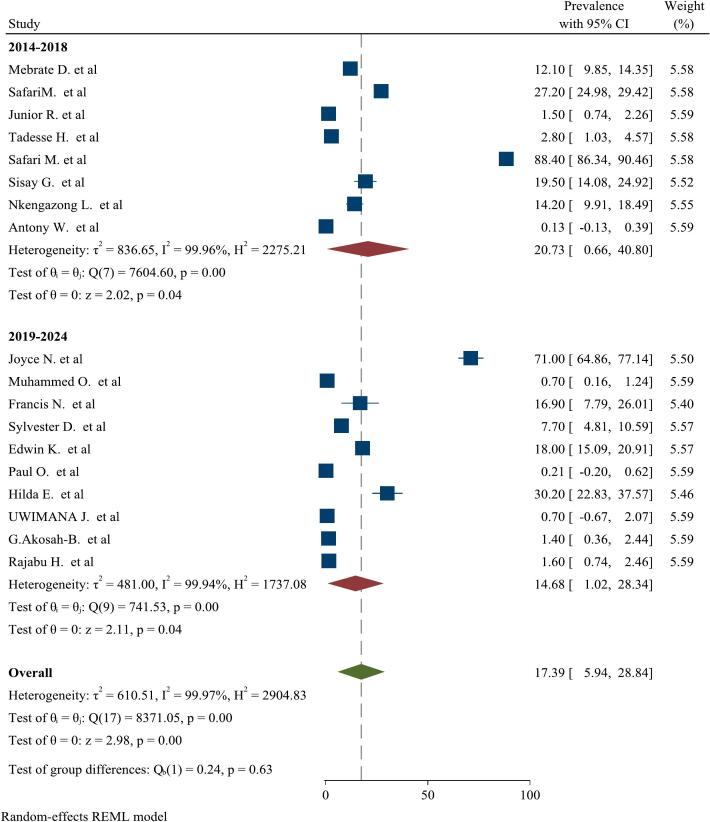
Subgroup analysis for pooled prevalence of malaria and *S. mansoni* coinfection based on year of publication from 2014 to 2024.

**Fig. 4:**
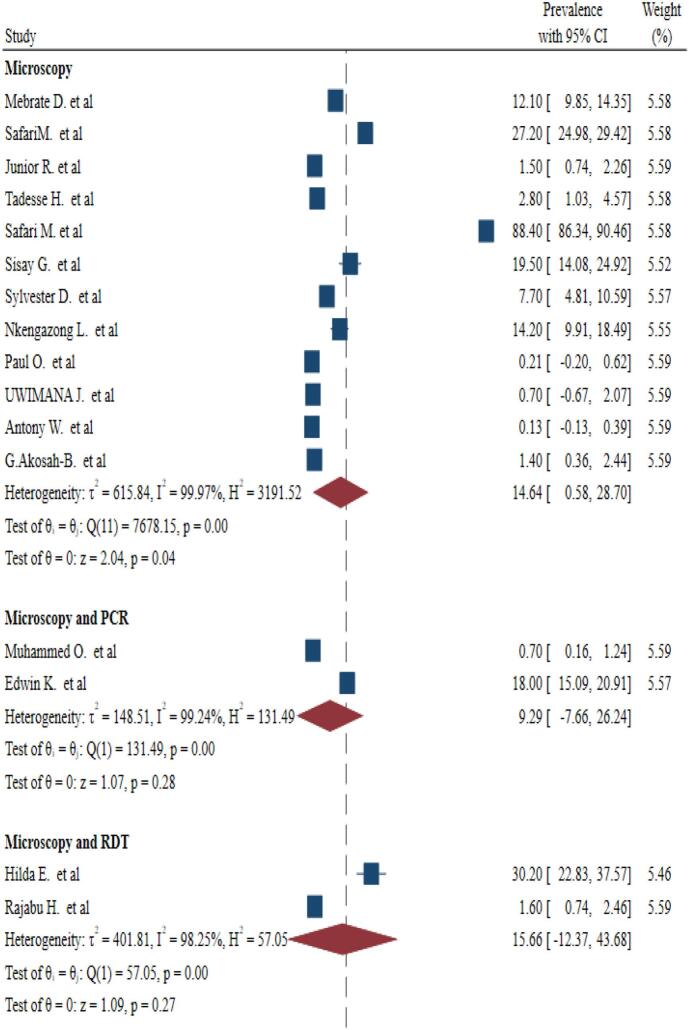

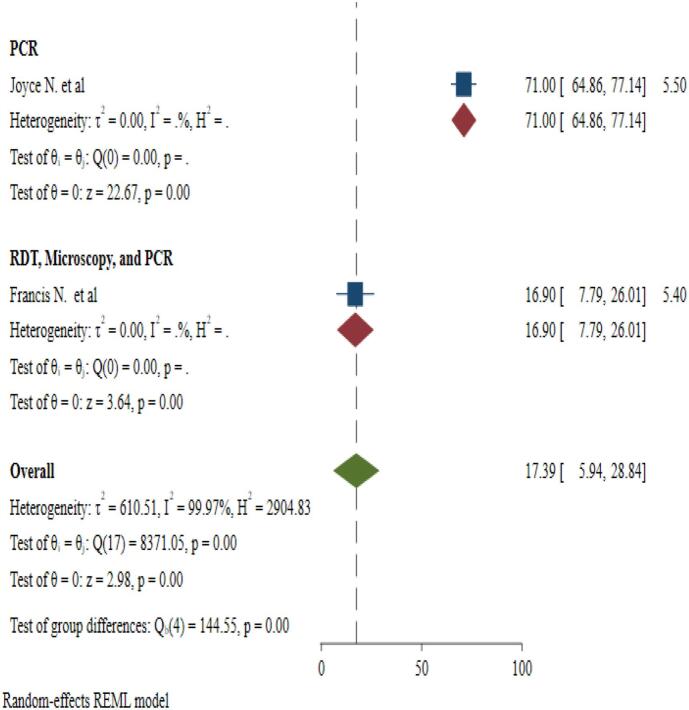
Subgroup analysis for pooled prevalence of malaria and *S. mansoni* coinfection based on diagnostic techniques.

There was high significant heterogeneity among included studies. Inverse of variance (I^2^) statistics showed greater than or equal to 99.97 % heterogeneity among studies for prevalence of malaria and *S. mansoni* coinfection. To identify the possible source of heterogeneity, subgroup analysis was performed for prevalence of malaria and *S.mansoni* coinfection on year of publication and diagnostic techniques. This meta-analysis showed no significant difference in frequency of malaria and *S. mansoni* coinfection among studies on year of publication. Also, the meta-analysis showed significant difference in frequency of malaria and *S. mansoni* coinfection among studies on diagnostic technique (Figs. 3 & 4).

### 3.6 Systematic review on associated factors of malaria and *S. mansoni* coinfection

Based on this review the prevalence of malaria and *S. mansoni* coinfection was in Oromia Regional state, western Ethiopia (12.10 %) and Amhara Regional State, Northwest Ethiopia (2.8 %,19.5 %) (Dufera et al., 2016; Hailu et al., 2018; Getie et al., 2015). Likewise, the frequency of malaria and *S. mansoni* coinfection in communities along the Albert Nile in Pakwach District in North Western Uganda, Kiryandongo district, Mid-Western region of Uganda, Kassena Nankana East Municipal East Region of Northern Ghana, and Ada East District of the Greater Accra region of Ghana was 71 %, 0.21 %, 7.7 %, and 1.4 %, respectively (Namulondo et al., 2024; Dassah et al., 2023; Oboth et al., 2019; Akosah-Brempong et al., 2021). Also, the prevalence of malaria and *S. mansoni* coinfection in Magu district, North-Western Tanzania, Mokali Health Areas of Democratic Republic of Congo, Saraya and Diourbel districts of Senegal, Busega district, Simiyu region, North-Western Tanzania, and Mara region, Northwestern Tanzania was 27.2 %, 1.5 %, 0.7 %, 1.6 %, and 88.4 %, respectively (Kinung'hi et al., 2014; Matangila et al., 2014; Afolabi et al., 2023; Kinung'hi et al., 2017; Mnkugwe et al., 2020). Moreover, based on this review the malaria and *S. mansoni* coinfection in sudano-guinean climatic zone of the North Region of Cameroon, Yoro village in Bokito town in Mbam and Inoubou Division of Center region of Cameroon, Kombewa, Kisumu Kenya, Bungoma Kenya, and Yorro Mbam Inoubou Center region of Cameroon was 16.9 %, 14.2 %, 18.0 %, 0.13 %, and 30.2 %, respectively (Kamau et al., 2021b; Lucia et al., 2015; Echelibe et al., 2019; Wekesa, 2018; Nkemngo et al., 2022b).

According to this review, the associated risk factors for malaria and *S. mansoni* coinfection are rural habitation, age, pregnant women in their second trimester, unemployment, drinking water source, mosquito nets, sanitation status, and health education (Wekesa, 2018). Furthermore, the prevalence of co-infections was significantly different based on gender, marriage, education, and body mass index categories (Kamau et al., 2021b). Moreover, living in residences with gaps between the walls and roofs, as well as regular water contact when swimming, were statistically significant risk factors for malaria and *S. mansoni* co-infection prevalence. Similarly, co-infections were more common in boys than in girls, and as age increased, infection prevalence reduced, with those aged 5–9 and 10–14 being more impacted than the elders one (Dufera et al., 2016; Namulondo et al., 2024; Afolabi et al., 2023; Kinung'hi et al., 2017).

## 4 Discussion

This systematic review and meta-analysis showed the prevalence of malaria and *S. mansoni* coinfection over a period of eleven years in sub Saharan Africa. In this systematic review and meta-analysis, the pooled prevalence of malaria and *S. mansoni* coinfection was 17.39 % (95 % CI: 5.94–28.84). This finding highlights a considerable health burden that affects treatment and control efforts, influencing global health policies aimed at lowering infectious illnesses. This figure aligns with the World Health Organization's goal for addressing co-infections, emphasizing the need of integrated disease management strategies and the allocation of resources for aiding vulnerable communities affected by both malaria and schistosomiasis. Understanding these prevalence is crucial to establishing targeted interventions in endemic regions, particularly for high-risk populations, with the ultimate goal of improving health outcomes and strengthening the effectiveness of World Health Organization's health policies (Duguay et al., 2024).

The high prevalence of malaria and *S. mansoni* co-infection complicates treatment regimens, diagnosis, and patient outcomes. One important difficulty is drug interactions between antimalarial drugs and schistosomiasis therapies, which can complicate treatment. Furthermore, finding adequate doses for coinfected individuals is problematic owing to changed pharmacokinetics, which frequently results in greater morbidity because these patients may have more severe symptoms requiring extensive treatment approaches (Mbah et al., 2014).

Furthermore, the overlap of symptoms between the two illnesses increases the likelihood of a misdiagnosis. Common malaria symptoms, such as fever and chills, are often mistaken with stomach discomfort and diarrhea caused by schistosomiasis, limiting diagnostic options. Standard tests may fail to detect both infections concurrently, especially in resource-limited settings, resulting in delayed treatment and additional worsening of patient health. The impact on patient outcomes is severe, since coinfection can considerably raise death rates due to the combined effects of both diseases. Patients frequently encounter chronic health problems, such as extended sickness, anemia, and organ damage. Furthermore, the complexity of treatment regimens not only raises healthcare expenses but also creates a significant load on healthcare systems, highlighting the critical need for new diagnosis and treatment procedures for coinfected individuals (Elden, 2010).

Likewise, this finding can strain healthcare resources, diverting attention, funding,and alter the host's immune response away from comprehensive malaria and schistosomiasis control programs. This can impede efforts to implement effective interventions, such as vector control and mass drug administration, ultimately impacting overall disease management.

This finding was inline with that reported in malaria and helminth co-infections endemic countries [6.7 %] (Afolabi et al., 2021), Ethiopia [18.4 %] (Abay et al., 2013), Tanzania [22.6 %] (Mazigo et al., 2010), Uganda [23.5 %] (Kabatereine et al., 2011), and Senegal [20.2 %] (Abugri et al., 2018). The similarities in findings regarding malaria and *S. mansoni* co-infections can be due to several common factors.This might be due to similar transmission dynamics, environmental factors, vector populations, human behavior, ecological settings, socioeconomic conditions, methodologies, susceptibility to malaria and *S. mansoni* co-infections, strategies for controlling malaria and *S. mansoni* such as vector control and mass drug administration. Correspondingly, this is supported by substantial amount of study which has been conducted on the immunological function of individuals who are co-infected with several parasites. It has been suggested that malaria may enhance a patient's vulnerability to *S. mansoni* infection (Sokhna et al., 2004; Briand et al., 2005).

However, this finding was higher than that reported in Kenya [0.1 %] (Brooker et al., 2012) and Uganda [2.1 %] (Brooker et al., 2012). This findings indicated that, in regions where malaria transmission is persistent, the interaction between malaria and *S. mansoni* may have decreased the population-level efficacy of malaria treatment in the absence of widespread praziquantel administration. Regardless of whether malaria transmission was high or low, co-infection with schistosomiasis caused the largest increase in per-person malaria episodes in areas with inadequate coverage of malaria therapy. Therefore, there is a chance to manage and take advantage of the resources and infrastructure already in place to maximize the effectiveness of the current malaria and schistosomiasis preventive and control programs for the population at risk that is found in this area. Additionally, looked into the integration of antimalarial medication administration and mass drug administration in schools, realizing the potential to coordinate efforts in one location for a population at risk. Furthermore, integrated non-pharmaceutical interventions like the supply of clean water, hygienic conditions, and proper sanitation that could significantly lower exposure to malaria and schistosomiasis infection (Midzi et al., 2011; Cohee et al., 2018).

This review found high heterogeneity in the prevalence of malaria and *S. mansoni* coinfection, with I^2^ statistics showing values more than or equal to 99.97 % at *P* = 0.00. To account for this heterogeneity, a subgroup analysis was conducted based on diagnostic approach and publication year. Even after subgroup analysis, there was still significant heterogeneity. This could be due to a variety of factors, including differences in environmental conditions, local healthcare practices, methodologies, sample sizes, socioeconomic status, diagnostic techniques, disease control measures, seasonal variations, and transmission dynamics, all of which could influence the reported incidence of coinfection across these studies (Goutam et al., 2007; Agasa et al., 2024; Duguay et al., 2023; Rouamba et al., 2019).

Moreover, differences in environmental conditions, healthcare infrastructure, and public health interventions play a crucial role in the observed heterogeneity of malaria and *S. mansoni* co-infection frequency. Variations in climate, geography, and water sources significantly influence the transmission dynamics of both diseases; for instance, areas with stagnant water facilitate mosquito breeding, thereby increasing the risk of schistosomiasis and leading to higher co-infection incidence. Regions with robust healthcare systems benefit from improved disease surveillance, diagnosis, and treatment capabilities, while those with limited access to healthcare often struggle to manage co-infections effectively, resulting in higher prevalence due to inadequate treatment and prevention measures. Furthermore, the effectiveness and reach of public health interventions such as vector control programs, mass drug administration, and educational campaigns vary widely. Comprehensive and well-implemented strategies can lead to lower co-infection prevalence, whereas regions lacking such interventions may experience higher rates due to increased exposure and untreated infections (Bisanzio et al., 2014; Nsubuga et al., 2011).

The presence of heterogeneity in the prevalence of malaria and *S. mansoni* coinfection caused by study populations due to variations in demographic, environmental, and socio-economic factors. Differences in age, gender, and health status can impact individual susceptibility and exposure levels to both infections. Additionally, socio-economic factors such as access to healthcare, sanitation, and education influence prevention measures and treatment adherence, further contributing to disparities in prevalence. Consequently, the interplay of these factors results in diverse patterns of malaria and *S. mansoni* coinfection across different populations (Mbah et al., 2014; Onkoba et al., 2015).

Study settings, including geographic location, environmental circumstances, and healthcare infrastructure, have a major impact on the variability of malaria and *S. mansoni* coinfection prevalence. Different exposure risks might result from urban *vs* rural settings. For instance, living close to freshwater sources may increase the levels of *S. mansoni* in rural regions, whereas population density and vector control strategies may have an impact on the dynamics of malaria transmission in urban areas. Furthermore, temperature and seasonal fluctuations can have an influence on malaria vector life cycles and schistosomiasis transmission. The availability and quality of healthcare services are also important, since improved access to diagnosis and treatment can lower infection rates. Overall, these different features within study contexts lead to observed differences in malaria and *S. mansoni* coinfection prevalence rates (Chiziba et al., 2024).

Malaria and *S. mansoni* transmission intensities have a considerable impact on the variability of coinfection prevalence, resulting in varied exposure levels within communities. Malaria transmission intensity is generally connected to variables such as numerous mosquito breeding sites and suitable meteorological circumstances, which can lead to an increase in malaria cases, potentially interfering with schistosomiasis transmission dynamics. Conversely, locations with high *S. mansoni* transmission, which are often linked with closeness to freshwater sources, might increase the risk of coinfection, particularly among populations involved in occupations such as fishing or agriculture. Since the presence of one disease may influence the susceptibility or immune response to the other, the interaction of these transmission intensities can produce complicated epidemiological patterns with varying prevalence rates in various populations and geographical areas. This dynamic therefore produces a patchwork of infection patterns that are impacted by the severity of both illnesses' local transmission (Viney and Graham, 2013).

However, this systematic review and meta-analysis showed that no significant difference in the prevalence of malaria and *S. mansoni* coinfection among studies on year of publication. This could be due to numerous factors, including epidemiological stability, methodological consistency, the effect of public health interventions, biological interactions, and potential publication biases, can be attributed for the lack of notable variations in the prevalence of malaria and *S. mansoni* coinfection across studies published over the years.

Furthermore, this systematic review and meta-analysis indicated that significant difference in the prevalence of malaria and *S. mansoni* coinfection among studies on diagnostic technique. The considerable disparity in the prevalence of malaria and *S. mansoni* coinfection observed in the systematic review and meta-analysis using diagnostic techniques can be attributed to a number of variables. These include diversity in diagnostic methodologies, a lack of standardization in diagnostic criteria among studies, and population differences.

The sensitivity and specificity of diagnostic procedures such as microscopy, PCR, and rapid diagnostic tests have a major impact on the heterogeneity of malaria and *S. mansoni* coinfection prevalence by altering detection rates and reporting accuracy. While microscopy is extensively employed, it may have reduced sensitivity, particularly in low-parasite-density infections, which might contribute to underreporting of malaria cases. In contrast, PCR has greater sensitivity and specificity, enabling for the identification of both malaria and *S. mansoni* at lower infection levels, revealing a more realistic prevalence of coinfection. Rapid diagnostic tests produce quick findings, but their sensitivity varies depending on the specific antigen targeted, which might influence the diagnosis of malaria cases, especially in mixed infections. Variability in diagnostic performance between contexts might cause inconsistencies in prevalence estimates, since certain groups may be underdiagnosed or misclassified, adding to the observed variation in malaria and *S. mansoni* coinfection rates. Finally, the selection and accuracy of diagnostic procedures influence our understanding of the epidemiology of these illnesses (Opoku Afriyie et al., 2023).

Variations in the prevalence of malaria and *S. mansoni* coinfection have been largely explained by changes in public health policies and diagnostic techniques over the years under study. The introduction of more sensitive diagnostic techniques, like PCR and rapid diagnostic tests, has improved the detection of low-density infections, which has led to more accurate prevalence estimates and better coinfection identification. Concurrently, improved surveillance systems and integrated control programs for both illnesses have enabled more effective monitoring and intervention tactics. Increased financing for public health efforts has also increased access to diagnoses and treatment, while environmental and behavioral measures aiming at lowering mosquito breeding and improving water cleanliness have had a significant influence on transmission dynamics. These discoveries are consistent with a larger epidemiological trend toward integrated disease treatment, highlighting the importance of comprehensive regimens that target many illnesses at the same time in order to attain long-term health outcomes (Afolabi et al., 2023; De Boni et al., 2021).

Based on this review malaria and *S. mansoni* coinfection was high prevalent in Mara region, Northwestern Tanzania (88.4 %) (Kinung'hi et al., 2017). This might be due to a combination of ecological and socioeconomic factors which cause high prevalence of malaria and *S. mansoni* coinfection in Mara region's. Its abundant freshwater bodies, including lakes and rivers, provide ideal breeding grounds for malaria-carrying mosquitoes and the freshwater snails that host *S. mansoni*. Furthermore, because the warm and humid conditions enhances the lifecycles of both the malaria parasite and *S. mansoni*, transmission rates are greatly increased. Additionally, socioeconomic challenges such as limited access to healthcare, and inadequate sanitation exacerbate the risk of infection, leaving communities with few resources for effective prevention and treatment (Duguay et al., 2023; Quayle et al., 2010).

According to this review, the associated risk factors like residence, age, pregnant women, gender, unemployment, drinking water source, mosquito nets, sanitation status, and education status were statistically significant risk factors for malaria and *S. mansoni* co-infection prevalence. This finding was similar with that reported associated risk factors of malaria and *S. mansoni* co-infection prevalence in Gabon (M'bondoukwé et al., 2018). It is support by World Health Organization report (Prüss-Üstün et al., 2016) and by report which conducted in South Africa (Quayle et al., 2010).

### 4.1 Strength of this study

We followed a predetermined protocol for search strategy and data abstraction. We employed widely acknowledged methodologies for critical appraisal and evaluating the quality of individual studies.

## 5 Limitations of this study

Language bias is possible because the included study were only published in English. Also, this review includes study from specific countries due to a lack of literature from other countries, which may alter the representativeness of the findings.

## 6 Conclusion and recommendation

The findings of this systematic review and meta-analysis concerning malaria and *S. mansoni* coinfection in sub Saharan Africa revealed that high prevalence of malaria and *S. mansoni* coinfection, indicating a substantial challenge in managing infections caused by this coinfection in healthcare settings. The observed increase in prevalence of malaria and *S. mansoni* coinfection underscores the urgent need for enhanced infection control measures and strengthened surveillance systems to limit the spread of malaria and *S. mansoni* coinfection in sub Saharan Africa. Additionally, collaborative efforts at local, national, and international levels are warranted to address the multifaceted factors contributing to malaria and *S.mansoni* coinfection and mitigate its impact on public health.

## Consent for publication

Not applicable.

## Funding

This systematic review and meta-analysis was not supported by any organization or individual.

## Ethical approval

Not applicable to this systematic review and meta-analysis.

## Author's contribution

W.A led the systematic review and meta-analysis, article selection, data mining, data screening, data extraction, statistical analysis, manuscript preparation, and overseeing the study's conceptualization. W.A, G.K, and A.A performed a critical role in finding relevant papers, extracting data, doing statistical analysis, and assisting with manuscript preparation. W.A, T.M, and D·W conducted full-text screening. W.A, M.N, and B·K were involved in statistical analysis consultation of the overall process of this systematic review and meta-analysis. W.A, E.T, Y.G, and A.B involved in data mining, data extraction, statistical analysis, manuscript writing, editing, and ensuring accuracy and completeness. Additionally, all authors actively engaged in critically reviewing the study's progress, data analysis, and manuscript preparation, involved in the approval of the final manuscript for submission, thereby affirming their endorsement of its content and findings.

## CRediT authorship contribution statement

**Wagaw Abebe:** Conceptualization. **Birhanu Kassanew:** Formal analysis. **Tadesse Misganaw:** Methodology. **Agenagnew Ashagre:** Methodology. **Getinet Kumie:** Conceptualization. **Marye Nigatie:** Formal analysis. **Yalewayker Gashaw:** Formal analysis. **Ephrem Tamrat:** Formal analysis. **Dagmawi Woldesenbet:** Methodology. **Alembante Bazezew:** Data curation.

## Declaration of competing interest

The authors of this systematic review and meta-analysis declared that they have no conflict of interest.

## Data Availability

All relevant data are found within the manuscript.
